# Supplementary material for: A new comparative framework for estimating selection on synonymous substitutions
Source: bioRxiv. 2025 Feb 6:2024.09.17.613331. Preprint. [Version 2] doi: 10.1101/2024.09.17.613331 (PMC11838523; doi:10.1101/2024.09.17.613331)
Supplement: Supplement 1 [file media-1.pdf]

Supplement

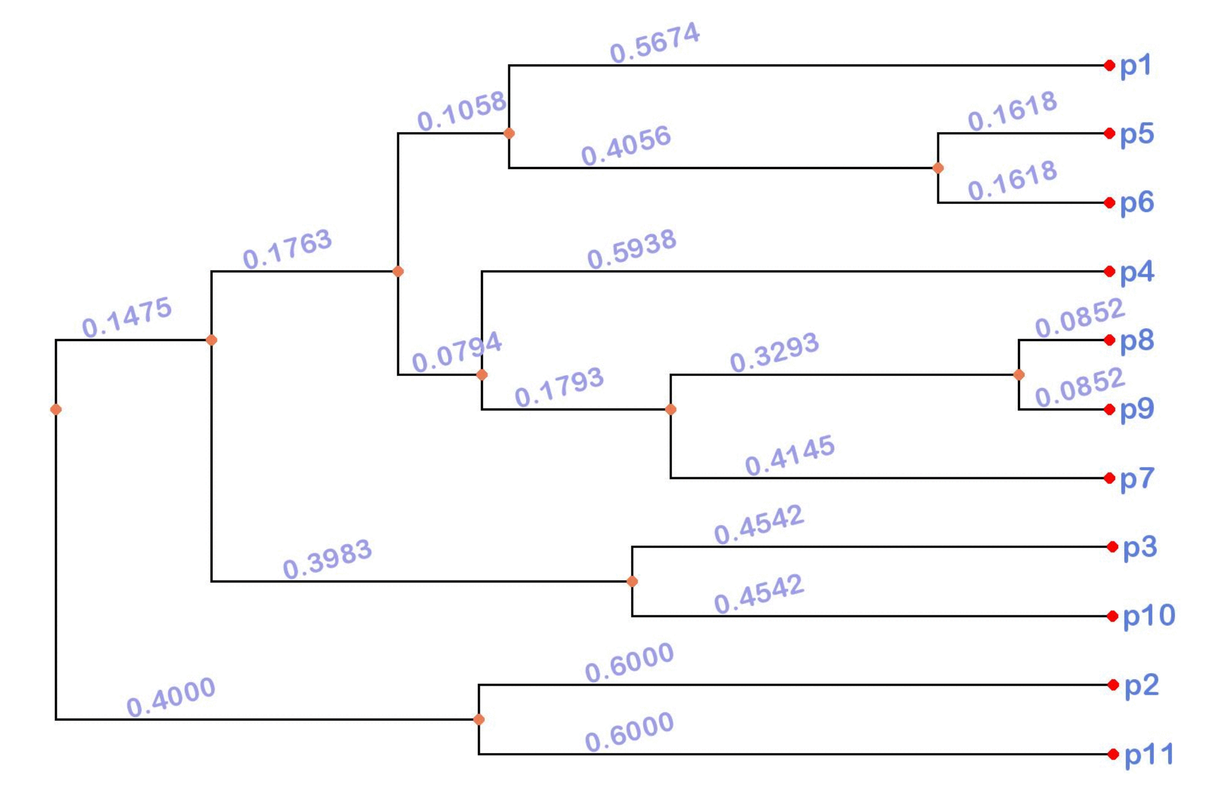

Figure S1. Phylogeny of population splitting events used by the forward simulator

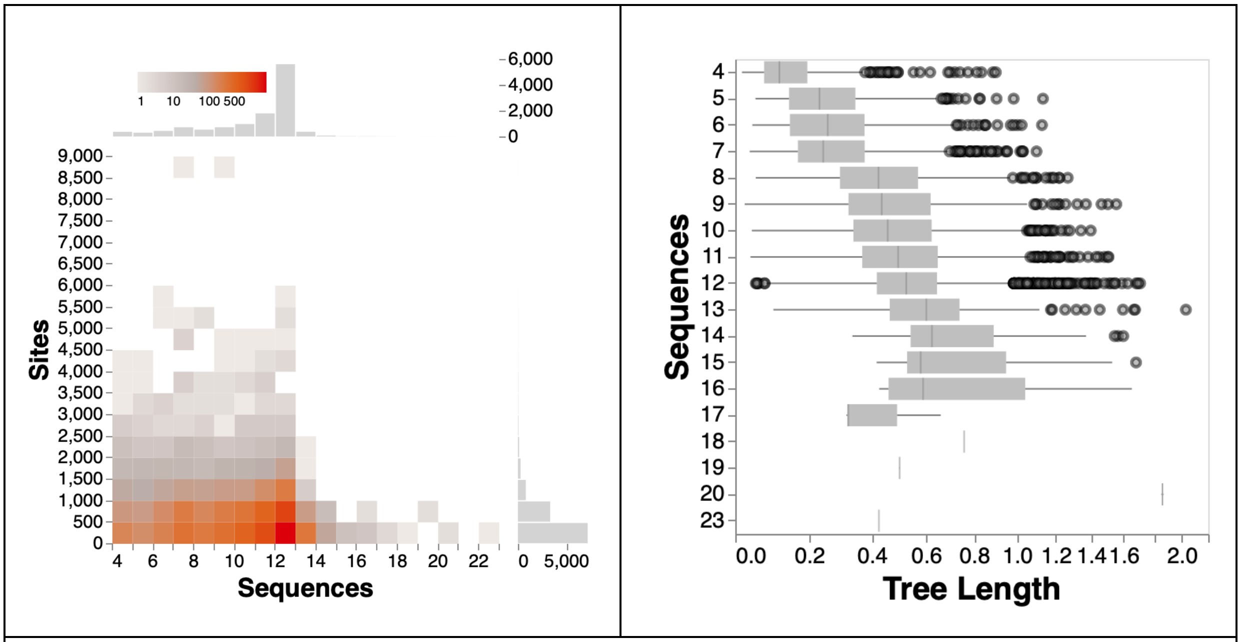

Figure S2. (Left) Dimensions of individual alignments. (Right) Tree lengths (expected substitutions / site) by the number of sequences.

Amino Acid	Neutral Set(s)
A	GCG GCT GCA GCC
C	TGT
C	TGC
D	GAC
D	GAT
E	GAA GAG
F	TTT TTC
G	GGA GGC GGT
G	GGG
H	CAC
H	CAT
I	ATT
I	ATC ATA
K	AAA
K	AAG
L	CTT CTA CTG CTC
L	TTG TTA
N	AAC
N	AAT
P	CCA CCC CCT CCG
Q	CAA
Q	CAG
R	CGA AGA AGG CGG
R	CGC CGT

S	AGT TCG TCT AGC TCA TCC
T	ACC ACT
T	ACG ACA
V	GTT GTC
V	GTA GTG
Y	TAC TAT

Table S1: True model for forward simulations. Each amino acid may have one or two classes of neutral synonymous substitutions, with each class' member codons represented by a separate row in the table. Substitutions within a class (row) are treated as neutral, synonymous substitutions between classes (rows) are treated as selected.

**Amino acid**	**Codon 1**	**Codon 2**	**Total subs**	**Per alignment**			**Alignments with non-zero counts**
				Mean	***Median***	***Maximum***	
**A**	GCA	GCC	59754	5.05	3	86	10328
**A**	GCA	GCG	58383	4.93	3	103	10030
**A**	GCA	GCT	34207	2.89	2	58	8729
**A**	GCC	GCG	52474	4.43	3	67	9822
**A**	GCC	GCT	114063	9.63	7	174	11044
**A**	GCG	GCT	30633	2.59	2	59	8627
**C**	TGC	TGT	55691	4.70	3	214	9450
**D**	GAC	GAT	160809	13.58	10	271	11257
**E**	GAA	GAG	178073	15.03	10	352	11278
**F**	TTC	TTT	151186	12.76	9	255	11165
**G**	GGA	GGC	79074	6.68	5	126	10598
**G**	GGA	GGG	46943	3.96	3	80	9618
**G**	GGA	GGT	48845	4.12	3	135	9597
**G**	GGC	GGG	30676	2.59	2	36	8779
**G**	GGC	GGT	107251	9.06	6	155	10931
**G**	GGG	GGT	17310	1.46	1	27	7083
**H**	CAC	CAT	78022	6.59	4	99	10381
**I**	ATA	ATC	41644	3.52	2	66	9319
**I**	ATA	ATT	43272	3.65	2	141	9057

**I**	ATC	ATT	120581	10.18	7	180	11079
**K**	AAA	AAG	150628	12.72	9	229	11198
**L**	CTA	CTC	25321	2.14	1	58	8102
**L**	CTA	CTG	104567	8.83	6	185	10822
**L**	CTA	CTT	24548	2.07	1	72	7431
**L**	CTA	TTA	24248	2.05	1	83	7173
**L**	CTC	CTG	85905	7.25	5	153	10515
**L**	CTC	CTT	73776	6.23	4	159	10470
**L**	CTG	CTT	59018	4.98	3	122	9945
**L**	CTG	TTG	159346	13.45	9	281	11225
**L**	TTA	TTG	39966	3.37	2	134	8460
**N**	AAC	AAT	137345	11.60	8	284	11159
**P**	CCA	CCC	48959	4.13	3	59	9848
**P**	CCA	CCG	77699	6.56	4	101	10398
**P**	CCA	CCT	25403	2.14	1	52	7612
**P**	CCC	CCG	51863	4.38	3	55	9832
**P**	CCC	CCT	59841	5.05	3	78	10073
**P**	CCG	CCT	25826	2.18	1	38	7930
**Q**	CAA	CAG	128038	10.81	7	247	10992
**R**	AGA	AGG	36025	3.04	2	73	8492
**R**	AGA	CGA	18976	1.60	1	40	6868
**R**	AGG	CGG	30640	2.59	2	55	8405
**R**	CGA	CGC	38502	3.25	2	58	9190
**R**	CGA	CGG	51359	4.34	3	68	9558
**R**	CGA	CGT	25308	2.14	1	43	7866
**R**	CGC	CGG	41889	3.54	2	49	9184
**R**	CGC	CGT	72197	6.10	4	109	10286
**R**	CGG	CGT	22725	1.92	1	26	7752
**S**	AGC	AGT	81440	6.88	4	143	10417
**S**	TCA	TCC	31573	2.67	2	57	8789
**S**	TCA	TCG	52827	4.46	3	107	9750
**S**	TCA	TCT	14331	1.21	0	44	5808
**S**	TCC	TCG	57298	4.84	3	82	9984
**S**	TCC	TCT	61194	5.17	3	125	10179
**S**	TCG	TCT	24585	2.08	1	51	7847
**T**	ACA	ACC	43368	3.66	2	63	9583

**T**	ACA	ACG	56548	4.77	3	84	9846
**T**	ACA	ACT	26158	2.21	1	64	7835
**T**	ACC	ACG	44578	3.76	2	65	9450
**T**	ACC	ACT	70086	5.92	4	109	10513
**T**	ACG	ACT	27903	2.36	1	55	8206
**V**	GTA	GTC	18135	1.53	1	34	7295
**V**	GTA	GTG	68488	5.78	4	128	10356
**V**	GTA	GTT	20096	1.70	1	80	7120
**V**	GTC	GTG	60431	5.10	4	78	10160
**V**	GTC	GTT	58191	4.91	3	107	10320
**V**	GTG	GTT	56655	4.78	3	94	10129
**Y**	TAC	TAT	99082	8.37	6	168	10865

Table S2. Descriptive statistics for each of the 67 SNSS substitutions in the *Drosophila* dataset, sorted by the encoded amino-acid.

Genetic algorithm testing: parametric simulations under the MG94 (null) model

The genetic algorithm performance was tested on null data through simulation of 1500 alignments under the Muse-Gaut 1994 (MG94) model. We generated simulated alignments under the empirical tree topology for the *Enterobacteriaceae* atpD gene alignment, which contains 15 taxa. Subsequently, we ran the genetic algorithm on 25 bootstrap replicates, each consisting of 100 alignments. The selection of the “best” model, either the null model or any alternate models, was evaluated for each replicate using the Bayesian Information Criterion (BIC) calculated for each model during the genetic algorithm procedure. BIC scores for each replicate can be found in `GA_results_for_MG94_data.csv`.
